# Tumor necrosis factor receptor 2 in allergen tolerance: a perspective view

**DOI:** 10.3389/fimmu.2025.1613719

**Published:** 2025-06-19

**Authors:** Guillem Montamat-Garcia, Murilo Luiz Bazon, Agnieszka Demczuk, Cathy Leonard, Markus Ollert

**Affiliations:** ^1^ Department of Infection and Immunity, Luxembourg Institute of Health, Esch-sur-Alzette, Luxembourg; ^2^ Institute of Immunity and Transplantation, Division of Infection and Immunity, University College London, London, United Kingdom; ^3^ Faculty of Science, Technology and Medicine, University of Luxembourg, Esch-sur-Alzette, Luxembourg; ^4^ Department of Dermatology and Allergy Centre, Odense University Hospital, Odense, Denmark

**Keywords:** allergen tolerance, allergen specific immunotherapy (AIT), TNFR2, peripheral tolerance, non-canonical NF-κB signaling, TNF receptor, immune checkpoint

## Abstract

Central and peripheral tolerance are key to maintain immune homeostasis. Imbalance of these processes often leads to diseases such as allergy, cancer or autoimmune disorders. During the immune response to allergens, several regulatory immune cells play a role in the development of peripheral tolerance and maintenance of homeostasis by inhibiting the development of CD4^+^ type 2 helper T cells, impairing the production of pro-allergenic cytokines, reducing the activation of effector cells driving allergic inflammation and generating allergen-neutralizing antibodies. However, the precise mechanisms of how peripheral immune tolerance is effectively maintained in healthy people, but not in allergic patients are still not well understood. Immune checkpoints have recently been proposed as critical molecular pathways across diseases for understanding how the immune system maintains homeostasis in many pathologies such as cancer, type 1 diabetes, multiple sclerosis, Crohn’s disease and allergy, among others. Particularly in the context of allergy, in-depth studies on immune checkpoint pathways might lead to emerging therapeutic targets. Tumor necrosis factor receptor 2 (TNFR2) is a crucial protein involved in promotion, expansion and maintenance of immune tolerance, being suggested as a key target for the treatment of several immune-based diseases including allergy. Here, we review the involvement of TNFR2 in allergic inflammation and allergen tolerance, its structural properties, signaling pathways, and importance for immune tolerance as a common mechanism, with the focus on possible implications for novel immunomodulatory treatments of allergic diseases.

## 1 Introduction

In addition to orchestrating defense against pathogens, immune cells also induce and regulate tolerance to antigens through central or peripheral tolerance. Allergic diseases occur when the immune system of the host fails to develop or maintain peripheral tolerance towards a specific allergen, a harmless environmental molecule that is usually tolerated in healthy individuals (1). In this process, CD4^+^ regulatory T cells (Tregs) triggered in the periphery upon exposure to external antigens (2) are an important subset of CD4^+^ T cells that play a role in the development of allergen tolerance and in the maintenance of immune homeostasis, exerting their immunoregulatory role on different cells of the innate and adaptive immune system. Particularly in the context of Immunoglobulin E (IgE)-mediated allergy, Tregs inhibit the development of type 2 CD4^+^ T helper (Th2) cells and impair their cytokine production in response to allergens (3, 4) by a combination of soluble and cell-to-cell contact molecules such as interleukin (IL)-10 and IL-35, Transforming Growth Factor-beta (TGF-β), granzymes, T-lymphocyte-associated protein 4 (CTLA-4), programmed cell death protein 1 (PD-1) or T cell immunoglobulin and mucin domain-containing Protein 3 (TIM3) (5, 6). In addition to allergen-specific Tregs, Type 1 regulatory cells (Tr1) and regulatory innate lymphoid cells (ILCregs) secrete IL-10 and TGF-β, which contribute to the resolution of the inflammatory response by suppressing both adaptive immune cells such as allergen-specific-IgE^+^ B cells and Th2 cells, as well as the innate arm of the allergic response composed of type 2 innate lymphoid cells (ILC2), eosinophils, mast cells and basophils (7–11). B regulatory cells (Bregs) are another subset of immune cells that have an immunosuppressive role (12, 13) and contribute to the induction of allergen tolerance mainly through the secretion of immunomodulatory cytokines, such as IL-35, IL-10 and TGF-β, which among others modulate T follicular helper responses (14, 15). Bregs are also known to produce anti-inflammatory IgG4 antibodies, which prevent allergen-specific IgE from binding to the allergen during an allergic reaction (16). Dendritic cells (DCs), and particularly tolerogenic DCs (tolDCs) are the initiators of allergen-specific immune tolerance, as they process and present antigens to naive T cells that will ultimately differentiate to allergen-specific Tregs (17).

In allergic patients, Th2 cells demonstrate antigen specificity toward allergenic proteins; however, there is a lack of corresponding antigen-specific Tregs due to disparate protein recognition, preventing effective regulation and resulting in allergen-specific loss of tolerance in airway allergy (18). Although a wide range of symptomatic pharmacotherapies are available that provide temporary and partial relief in the majority of allergic patients, their side effects as well as the necessity to use them for lengthy periods of time with no perspective of permanent cure, have prompted the will to develop improved allergen-specific immunotherapies with the prospect of sustainable restoration of tolerance (19, 20). Allergen-specific immunotherapy (AIT) is the only curative approach with long-lasting disease-modifying properties. AIT consists of delivering increasing doses of the causative allergen over time to reactivate immune tolerance and achieve clinical non-reactivity to the allergen. While the long-term immunological consequences correlating with clinical response and cure from allergy in AIT are well-defined (21–23), the molecular mechanisms that initiate and promote the switch from pathologic Th2 immunity to an allergen-specific immune tolerance are still poorly understood.

Recently, immune checkpoint receptors have been postulated as crucial players in recognizing how the immune system can reestablish peripheral tolerance and suppress allergic immune responses (24–27). However, findings and application of these molecules in the field of allergy are not as advanced as in other areas of clinical research such as cancer immunotherapy, type 1 diabetes (T1D), multiple sclerosis (MS), and other autoimmune diseases. Similar to allergy, many of the strategies to treat these diseases, except for cancer therapy, rely on the re-induction of central or peripheral tolerance. Indeed, there are common but unexploited mechanisms across immune-based diseases that cannot be disregarded when studying a specific pathology. Tumor necrosis factor receptor 2 (TNFR2) has been extensively investigated in other immune disorders that share the finding of dysregulated immune tolerance with IgE-mediated allergic diseases. TNFR2 overexpression (28, 29) in tumors suppresses anti-tumor immunity (30–32). In autoimmune diseases, TNFR2-expressing Tregs dampen inflammation in T1D (33), while in MS the receptor engagement supports oligodendrocyte-mediated immune modulation (34–36). TNFR2 has also been shown to ameliorate graft-versus-host disease in hematopoietic stem cell transplantation (37) and enhance allogeneic transplant tolerance (38). Although these studies underscore the immunoregulatory/anti-inflammatory properties of TNFR2, in certain circumstances, TNFR2 engagement can also drive inflammatory responses in a context-dependent role by inducing pro-inflammatory cytokine production, enhancing cytotoxic functions and reinforcing effector T cell activity (39–43).

Despite its proven potential in the context of multiple immune-mediated diseases, TNFR2 has not been deeply investigated for improving the treatment of allergy. Here we review the role of TNFR2 as a prominent actor for immune tolerance across diseases, with an emphasis on IgE-mediated allergy. Understanding the mechanisms behind this receptor and its implications as a potential immunomodulator for allergy treatment may lead to new ways of improving AIT.

## 2 Structure and multifaceted tuning of TNFR2

Tumor Necrosis Factor (TNF) is a pleiotropic cytokine of the TNF ligand superfamily (TNFSF) which exerts its biological effects via its two structurally related but functionally distinct receptors, TNFR1 (also known as TNFRSF1A, CD120a or p55) and TNFR2 (TNFRSF1B, CD120b or p75). Through the interaction with these membrane-anchored receptors, TNF has been shown to support two paradoxical mechanisms, a pro-inflammatory (via TNFR1) and an anti-inflammatory (via TNFR2) signaling axis, thus being involved either in the development of different autoimmune inflammatory diseases (e.g., psoriasis, rheumatoid arthritis) or, on the opposite, in the dampening of the immune response (e.g., cancer) (34, 44, 45).

**Figure 1:**
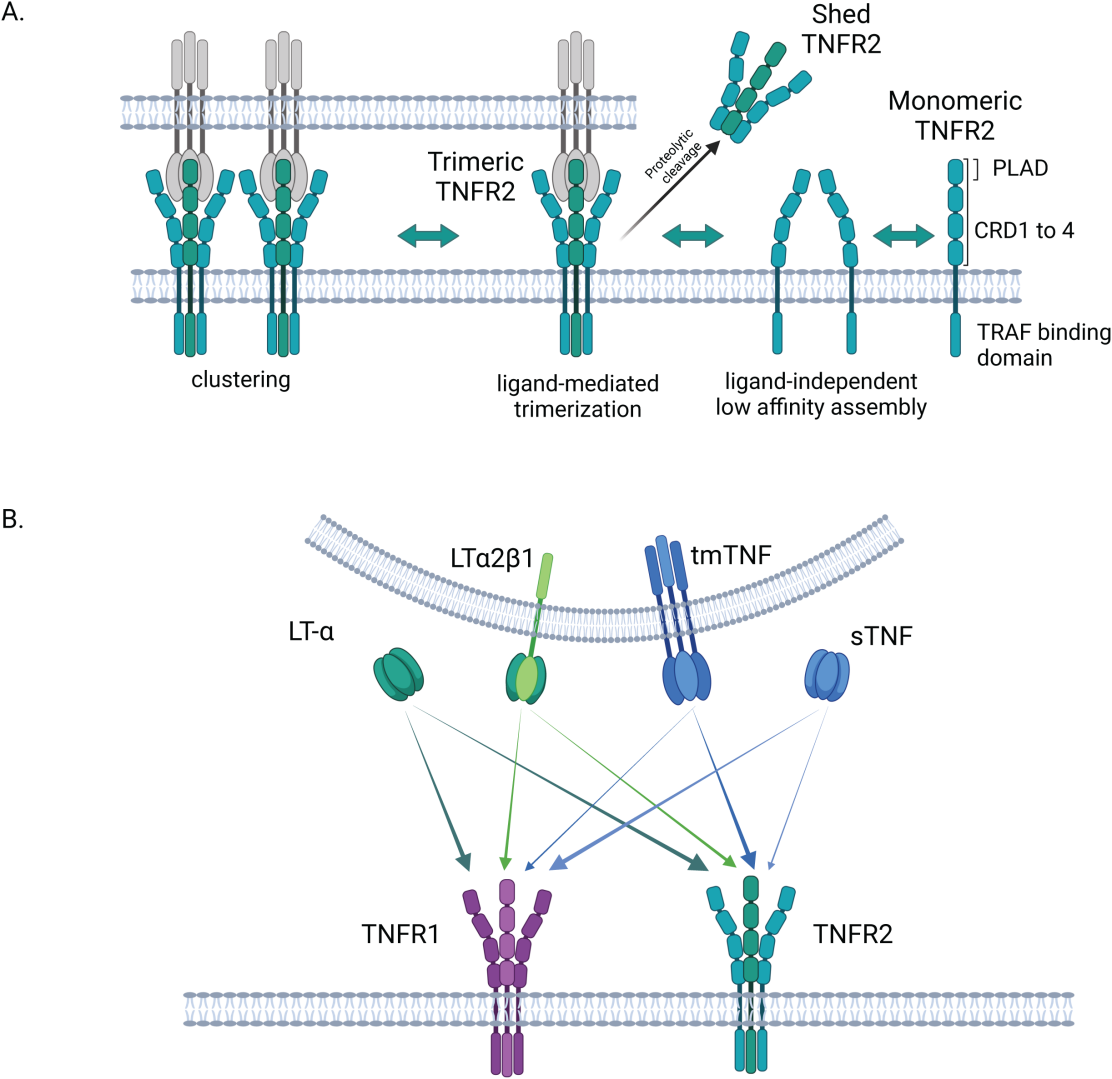
Structure and activation of TNFR2. **(A)** Structure and assembly of TNFR2: in its extracellular N-terminal region, TNFR2 exhibits 4 cysteine rich domains (CRDs). Pre-ligand assembly domain (PLAD) in CRD1 is involved in the formation of low-affinity self-complexes. The binding to a soluble or membrane-bound ligand triggers the trimerization of the receptor. Once trimerized, TNFR2 is proposed to associate in clusters at the cell surface. In its trimeric form, TNFR2 can be shedded by proteolytic enzymes in a soluble form. **(B)** TNFR2 activation by different ligands and comparison to TNFR1: TNFR2 is more strongly activated by the transmembrane form of TNF than by its soluble form, contrary to TNFR1. LT-α is another high affinity ligand for TNFR2 (and TNFR1) in its soluble homotrimeric form. The membrane-bound heterotrimer LTα2β1, composed of two LT-α and one LT-β monomers has been recently shown to equally activate both, TNFR1 and TNFR2. Created in BioRender. Demczuk, **(A)** (2025) https://BioRender.com/z93r097.

Just as ligands of the TNF superfamily, which display structural similarities notably in their TNF homology domain (THD), TNF receptors also exhibit some common traits (46). The THD mediates the trimerization of the ligands, thereby forming groove-shaped connection areas essential for interactions with the receptors of the TNF receptor superfamily (TNFRSF) (47). The latter are characterized by the presence of 2 to 4 cysteine-rich domains (CRDs) in their N-terminal extracellular region (**Figure 1**). TNFR1, as TNFR2, includes four CRD domains which play a role either in the formation of TNFR self-complexes or as ligand-binding domains (48).

According to the structures and functions displayed by their C-terminal intracellular region, members of the TNFRSF can be divided in 3 groups: 1) decoy receptors that lack a cytosolic domain and are devoid of signaling capacities; 2) death receptors which, like TNFR1, have a cytoplasmic death domain (DD) that enables most death receptors to induce apoptosis of cells; 3) TNF-receptor associated factor (TRAF)-interacting receptors (including TNFR2), that harbor cytoplasmic domains most often engaged in direct interactions with TRAF adapter proteins, inducing pro-inflammatory signaling pathways and promoting cell survival and tissue regeneration, but lacking a DD (41, 46, 49).

The assembly of TNFR2 on the cell surface encompasses different levels. At first, membranous TNFR2 can form self-complexes on the cell surface, based on a ligand-independent mechanism requiring interactions of the N-terminal pre-ligand assembly domain (PLAD) in CRD1 of the protomers (41, 48, 50). Then, according to the ligand-mediated trimerization model of TNSFR receptor activation, the binding of a soluble or transmembrane TNFSF trimer, through interactions between THD and CRDs 2 and 3, triggers the trimerization of the receptor proteins (45) (**Figure 1A**). These interactions, requiring a double trio of receptor proteins and of ligand molecules, are thought to induce a conformational change in the TNFR that is at the origin of the activation of the signaling cascade (48, 50). In addition, it was also suggested that TNF-TNFR2 double trimers are involved in the editing of complexes, thus further contributing to the activation of the signaling pathways (41, 51). Such complexes can combine to higher order aggregates or clusters at the cell surface that maximize cell signal initiation (47). The intracellular domain of TNFR2 is involved in such multi-dimensional networks via its interactions with TRAF2 (48). Finally, as TNFR1, TNFR2 can be proteolytically processed in soluble forms capable of decoying the TNF ligands (52, 53) (**Figure 1A**).

In contrast to the ubiquitously expressed TNFR1, TNFR2 is typically present at low levels on several cells of the immune system such as myeloid cells, Natural Killer (NK) cells, T and B cells, but also on endothelial cells (54). Thus, these cells express both receptors in a dynamic fashion that varies with the immune cell environment. This co-expression may have a critical role to play in the balance between signaling pathways controlling apoptosis and cell proliferation (55).

While TNFR1 is equally activated by both the trimeric soluble and membrane bound forms of TNF, TNFR2 exhibits a stronger binding reaction with transmembrane TNF (tmTNF) (56). The differences in these TNF/TNFR interactions result in a modulation of the Nuclear factor-κB (NF-κB) activation, as explained in the subsequent section. Besides TNF, Lymphotoxin-α (LT-α) is another ligand for both TNFR1 and TNFR2 (56, 57). LT-α, also known as TNF-β, is structurally similar to TNF. Compared to TNF, LT-α is secreted by a more limited range of immune cells (CD4^+^ and CD8^+^ T cells, B cells, NK cells and Lymphoid Tissue Inducer cells) (58, 59). While it has long been known that this sometimes-neglected TNF ligand can interact with high affinity with both receptors (TNFR1 and TNFR2) through its soluble homotrimeric conformation (LT-α3), it was recently shown that these receptors can be activated in an alternative way by heterotrimers formed by the aggregation of two LT-α subunits with one membrane-bound Lymphotoxin-β (LT-β), in a LTα2β1 heterotrimer configuration (50). Heterotrimeric LTα2β1, but not its predominant counterpart LTα1β2, the established ligand of Lymphotoxin-β receptor (LTBR), is an activator of TNFR1 and TNFR2 (50) (**Figure 1B**).

Besides its activation by the aforementioned ligands, it has been shown that through binding to tmTNF, TNFR2 can in turn trigger the expression of soluble TNF in a forward and reverse crosstalk between both TNF receptors (60). In addition, shed soluble TNFR2 is likely involved in the regulation of immune cells and inflammatory responses through neutralization and consequent inhibition of soluble TNF, and possibly also other TNFSF ligands (52, 53). In addition, TNFR2 may have regulatory effects on TNFR1 signaling by the sequestration of TRAF2 via interaction with its intracellular domain, making this factor less available for initiation of the TNFR1-induced TRAF2-TNFR1 associated death domain protein (TRADD) step (61). Such a feature of TNFR2 shows yet another mechanism by which this receptor prevents and reduces excessive immune activation and inflammatory processes such as those observed in allergic diseases.

## 3 Signaling pathway of TNFR2

**Figure 2:**
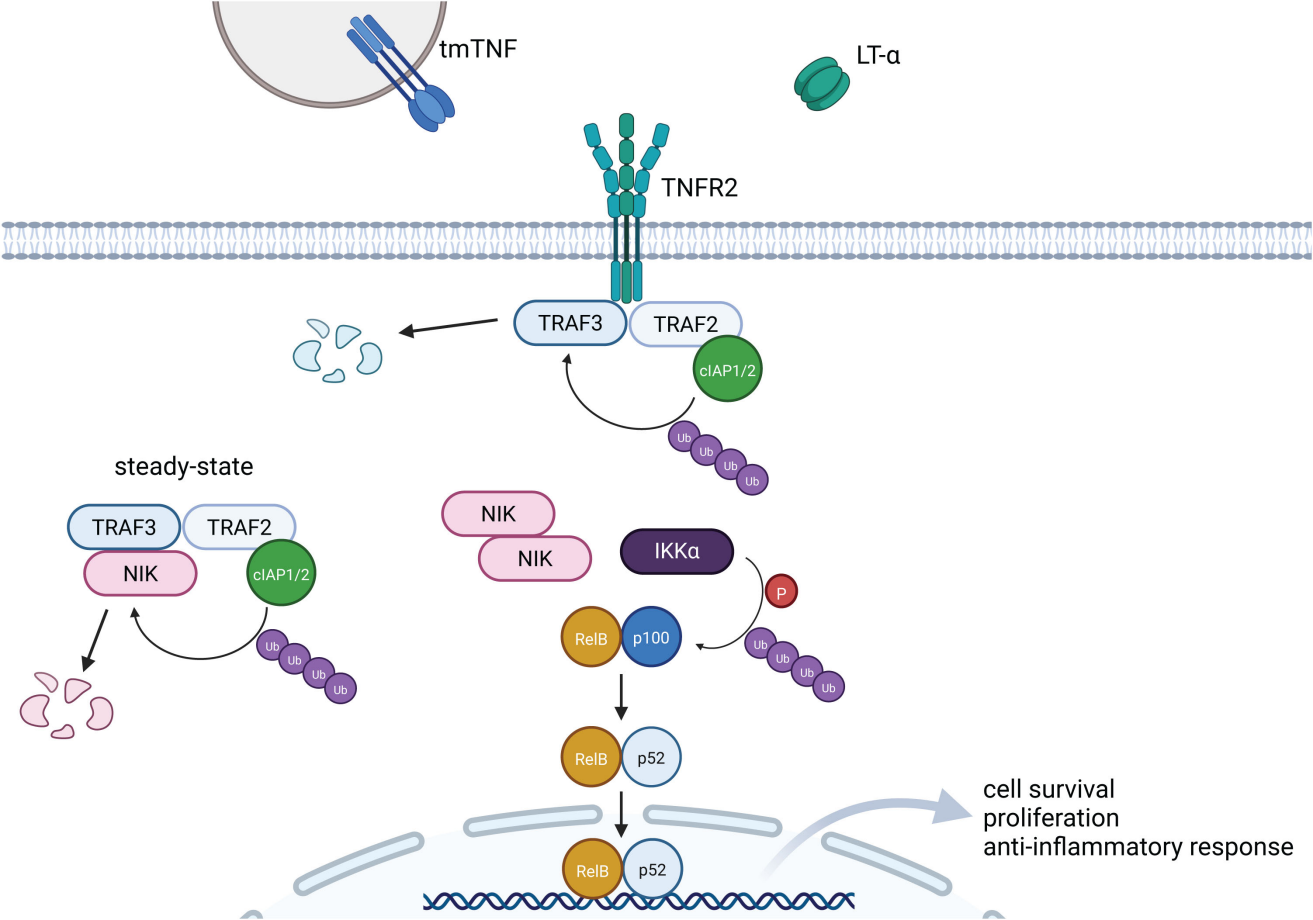
Signaling pathway induced by TNFR2. Signaling induced by TNFR2 results predominantly in the activation of the non-canonical NF-kB pathway. Upon binding of the ligand, NIK is released from TRAF3-TRAF2-cIAP1/2-dependent ubiquitination and degradation. Accumulation of NIK and subsequent activation of IKKα induces phosphorylation and ubiquitination of p100, which results in generation of p52. Finally, p52-RelB heterodimer translocates to the nucleus and targets transcription of genes associated with cell survival and anti-inflammatory response. Created in BioRender. Demczuk, A. (2025) https://BioRender.com/h10c322.

Activation of several members of the TNF receptor superfamily, including TNFR2, leads predominantly to the induction of non-canonical NF-κB signaling (62). Ligand binding to TNFR2 results in formation of the TNFR2 trimer and recruitment of adaptor proteins such as TRAF2, TRAF1/3 and Cellular Inhibitor of Apoptosis Protein 1/2 (cIAP1/2). In steady-state conditions, these adaptor proteins form a complex with NF-κB inducing kinase (NIK), the central regulator of non-canonical NF-κB signaling (**Figure 2**). This leads to ubiquitination and proteasomal degradation of NIK, and prevents activation of the NF-κB pathway. Upon TNFR2 activation, this complex is disrupted as TRAF3 undergoes ubiquitination by E3 ubiquitin ligase cIAP1/2 and subsequent degradation. This results in accumulation of NIK and activation of Inhibitor of NF-κB (IκB) kinase α (IKKα). Consequently, p100 is phosphorylated and processed to form p52, which generates active p52/RelB heterodimers (**Figure 2**) (63, 64). Upon translocation to the nucleus, p52/RelB (also known as non-canonical NF-κB) stimulates the expression of genes associated with cell survival, proliferation and lymphoid organ development (65, 66).

To a lesser extent, TNFR2 signaling can also result in the activation of canonical NF-κB pathway (45, 67). In this case, TRAF2 and cIAP1/2 mediate the recruitment of TGF-β activated kinase 1 (TAK1) and NF-κB essential modulator (NEMO) adaptor proteins, which leads to NEMO-dependent IκB kinase β (IKKβ) activation. Then, IκB is phosphorylated by IKKβ, and degraded in ubiquitin-dependent manner (68). As a consequence, p50/p65 heterodimers are released from IκB inhibition, translocate to the nucleus and activate the transcription of genes related to inflammatory response (69). Activation of the canonical NF-κB pathway is quick, transient and induced by multiple stimuli such as pathogen associated molecular patterns (PAMPs), damage associated molecular patterns (DAMPs), proinflammatory cytokines, and DNA damage via the engagement of various receptors: TNFR1, T cell receptors (TCRs), and Toll-like receptors (TLRs) (63, 70). It results in the initiation and development of inflammation, for example by promoting macrophage differentiation towards M1 phenotype (71), mediating induction of the Th1 (72) and Th17 (73) lymphocyte subsets or providing the priming signal for NLRP3 inflammasome activation (74). Conversely, activation of the non-canonical NF-κB pathway is occurring in a slow, persistent (45) and restricted manner – it is induced only by stimulation of specific receptors belonging to the TNFR superfamily: TNFR2, B-cell activating factor receptor (BAFFR), CD40, LTβR, Receptor activator for NF-κB (RANK), and OX40, among others (65). This signaling axis is involved in the development of regulatory immune cells and lymphoid organs. Thus, signaling induced by activation of TNFR2 can be considered mostly as anti-inflammatory, promoting immune regulation, immune tolerance and development of immune cells. Indeed, over-activation of TNFR2 and non-canonical NF-κB pathways in T cells can lead to Sézary syndrome (75), a systemic type of skin T cell lymphoma characterized by a strong immune suppressive capacity (76). Therefore, tuning and modifying the TNFR2 intracellular signaling cascade could support a novel therapeutic strategy to reestablish peripheral tolerance towards allergens as it can have crucial effects when engaged in the appropriate immune cell type.

## 4 TNFR2 as modulator of immune cells

As described above, TNFR2 downstream signaling drives mainly the activation of the non-canonical NF-κB complex as well as under certain circumstances the canonical NF-κB pathway. Since these two signaling avenues are common in immune cells and drive their proliferation and activation, engaging or blocking TNFR2 can have a substantial impact on immune responses.

**Figure 3:**
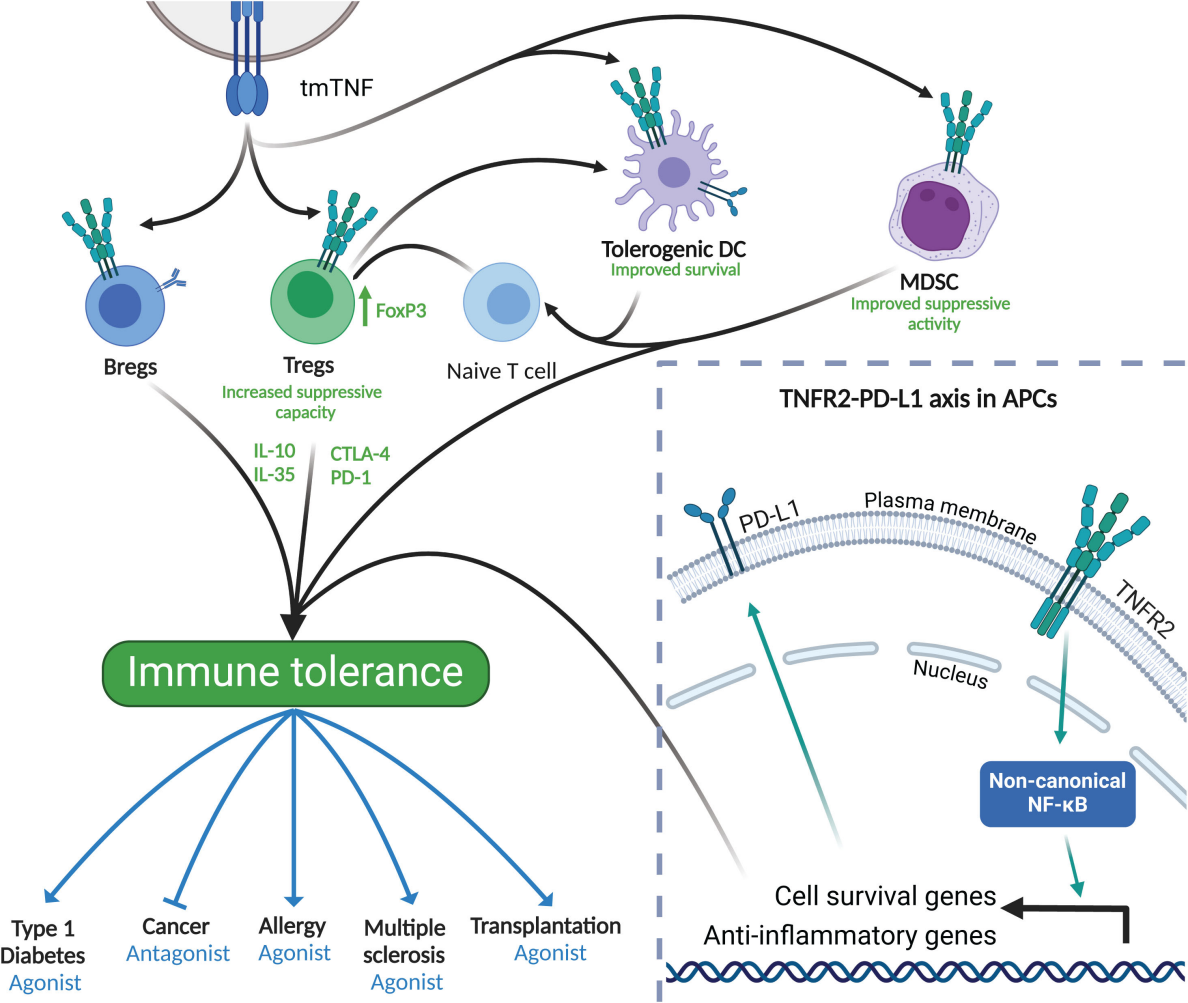
TNFR2 in immune tolerance. Bottom right frame: TNFR2 activation and downstream signaling leads to the activation of anti-inflammatory and cell survival genes. Of importance, the expression of PD-L1 is crucial for tolerance induction in APCs. Main frame: TNFR2 is expressed in a wide variety of cells including DCs, MDSCs, Tregs and Bregs. When activated, mainly through tmTNF, these cells increase their anti-inflammatory capacity contributing to immune tolerance to both, auto- and environmental antigens, including allergens. Immune tolerance induced by TNFR2 expressing cells can be modulated using either TNFR2 agonists or antagonists to stimulate or dampen the immune system as therapeutic strategies. Created in BioRender. Demczuk, A. (2025) https://BioRender.com/x12l712.

When T cells subsets become activated, an upregulation of several members of the TNFR superfamily occurs. Tregs in particular engage TNFR2, glucocorticoid-induced TNFR-related protein (GITR, TNFRSF18), OX40 (CD134, TNFRSF4) and Death receptor 3 (DR3, TNFRSF25) (38). Indeed, TNFR2 is mostly expressed on Tregs and, in both human and mouse, its expression identifies subsets that are maximally immunosuppressive (77) and expanded, show enhanced activation, and have phenotypic stability (44, 77–79) (**Figure 3**). TNFR2 can also be found in activated conventional T cells (Tconvs), though at a lower level than in Tregs (80). CD4^+^ T cells with a genetic deletion of TNFR2 (TNFR2 knockout) showed an increased expression of RAR-related orphan receptor gamma (RORγt) and impaired IL-17 production, which depends on TNFR2-mediated NF-κB activation (81). Thus, TNFR2/NF-κB contributes to immune homeostasis by increasing FoxP3 expression in Tregs, while inhibiting RORγt expression in Th17 cells (30). In the same line, using the TNFR2 antagonist Etanercept and TNFR2 gene deletion by CRISPR/Cas9, Skartsis et al. (82) reported a decrease in TNFR2 expression and reduction in CD25, FoxP3 and HELIOS expression ex-vivo in human Tregs, thus confirming the role of TNFR2 in promoting the proliferation of Treg and maintaining their lineage. Together, these studies corroborate, through clinical and preclinical findings, that the TNFR2 pathway is a key factor in the maintenance of FoxP3 expression and sustained function of Tregs, a fundamental feature for the maintenance of peripheral tolerance not only to autoantigens, but also to environmental allergens.

Aside from T cells, other immune cells such as B cells, NK cells and some DC subtypes express TNFR2 (**Figure 3**), particularly after stimulation (83–85), granting them unique features upon receptor engagement. In the context of tolerance, TNFR2 was shown to be expressed in B cells, where it correlates with their expression of IL-10, which is characteristic of regulatory B cell properties (83) (**Figure 3**). Along the same line, recent evidence shows that TNFR2 is associated with Bregs featuring a memory phenotype (86). Interestingly, although TNFR2 expression is associated with IL-10 production in human B cells, the suppressive capacity of memory B cells expressing TNFR2 could not be attributed to a soluble factor (86). This indicates that B cells expressing TNFR2 could have potent cell-to-cell contact inhibitory and regulatory functions. The role of TNFR2 in NK cells is still elusive since different studies report opposite functions of NK cell biology associated with this receptor. On the one hand, TNFR2 has been associated with an increased production of interferon gamma (IFN-γ) by NK cells, but on the other hand, it has also been linked with loss of NK cell cytotoxic capacity promoted by the tumor microenvironment (87, 88). Furthermore, TNFR2 has been shown to be pivotal in the interaction between NK cells and DCs through the expression of tmTNF by DCs, thus primarily activating TNFR2 but not TNFR1 on the surface of NK cells (89). Besides the activation of NK cells via tmTNF expressed by DCs, TNFR2 is also important for DC biology itself. TNFR2 signaling through the non-canonical NF-κB pathway has been described as an enhancer of DC survival (90, 91) (**Figure 3**). Monocytes, like DCs, also express TNFR2, at high levels particularly in non-classical monocytes (92, 93). It was shown that autocrine engaging of TNFR2 by human monocytes upregulates the anti-inflammatory cytokine IL-10, without promoting a simultaneous inflammatory phenotype (94). Like DCs, Myeloid-derived suppressor cells (MDSCs) require membrane TNFR2 expression for exerting their suppressive activity (**Figure 3**) (95).

In addition to promoting survival and enhancing suppressive functions, TNFR2 is also known to be tightly linked to the expression of other key molecules that are relevant for immune tolerance such as programmed death-ligand 1 (PD-L1) (**Figure 3**, enlarged window). In fact, activation of the TNFR2-induced non-canonical NF-κB pathway leads to an increase, among others, of PD-L1 in antigen presenting cells (96, 97), enhancing cell survival via the TNFR2–PD-L1 axis. Hence, TNFR2 activation can induce long-lived tolerogenic DCs expressing PD-L1, which would be crucial for peripheral tolerance-inducing therapies such as allergen-specific immunotherapy (**Figure 3**).

In summary, TNFR2 has been shown to be able to modulate a plethora of immune cells pivotal for the induction and maintenance of immune homeostasis (**Figure 3**). Therefore, designing new therapeutics intervening with the TNFR2 pathway in these cell types will be one of the keys to optimizing treatments that can effectively restore peripheral immune tolerance to allergens through antigen-specific approaches.

## 5 Mechanisms and challenges of allergen-specific immunotherapy

Allergic diseases are caused by a defective peripheral immune tolerance towards allergens, non-harmful molecules that induce strong immune responses in allergic individuals, and are characterized by a variety of clinical symptoms such as rhinitis, conjunctivitis, cough, breathlessness, allergic shock (anaphylaxis), skin rash or diarrhea (98, 99). At the cellular level, allergy is marked by a skewed immune imbalance towards a Th2 type response (100), coupled with insufficient antigen-specific Treg activity (18). As a consequence, allergen specific IgE antibodies are induced, bound to the surface of mast cells, eosinophils and basophils which upon binding with the allergen trigger the hallmark clinical symptoms of allergy (100).

Various symptom-relieving medications such as antihistamines, corticosteroids, leukotriene receptor antagonists (101) or more recently Type-2 immune pathway targeting drugs like monoclonal antibodies or JAK/STAT pathway inhibitors (102–105) are used to alleviate symptoms of allergic patients – however, they do not have a long-term curative effect. Currently, the only treatment that can provide lasting tolerance towards allergens is AIT. It is based on administering increasing doses of the causative allergen and is able to generate immune tolerance overtime providing long-term effects (106, 107). Mechanistically, AIT induces suppression of Th2 responses (108), skewing the immune landscape towards Th1 cells (109). Importantly, this treatment also leads to generation of allergen specific regulatory cells such as Tregs and Bregs (106, 110, 111). Furthermore, AIT induces B cell class switching towards the production of protective IgA and IgG4 (111). AIT is also able to affect the innate immune system to prevent further skewing of naïve T CD4^+^ cells to a Th2 phenotype by modulating DCs (112) and ILC2s (113, 114).

Despite remaining the only curative option for allergies, AIT still faces unmet needs. While AIT to insect venom demonstrates an exceptional efficacy with a cure rate of over 90% (115), AITs with other allergens have not achieved comparable success. While these AITs alleviate symptoms and improve quality of life significantly, providing a sustainable cure in the vast majority of patients remains an unmet goal (116–118). Further challenges are related to potential side effects and the absence of robust biomarkers that could predict the outcome of AIT at an early stage (107, 119). Thus, it is of particular importance to deeply investigate and understand the mechanisms underlying successful AIT, search for immune signatures that characterize clinical non-responsiveness, and finally, identify immune checkpoints that could be targeted in order to improve the restoration of peripheral allergen tolerance.

## 6 TNFR2 as a potential target in allergy treatment and prevention

TNFR2 is a key molecule in peripheral immune tolerance due to its crucial role on many cell types with regulatory properties, such as Tregs and Bregs (**Figure 3**) (83, 120, 121). Thus, TNFR2 might be a relevant therapeutic target in maintaining and restoring allergen tolerance, thus helping to overcome some of the existing shortcomings of AIT in curing allergy. Indeed, as recently shown in a pre-clinical murine AIT model characterized by successful phenotypic and immunologic cure using therapeutic components at endotoxin-low conditions, thus resembling human AIT trials, the TNFR2 axis was upregulated early on during AIT in innate and adaptive immune cells as well as after completion of AIT in regulatory T cell subsets (122). In this study, an optimized dose of B-type CpG oligodeoxynucleotides (CpG-ODN), a TLR9 agonist, was evaluated as an immunoregulatory adjuvant modulating the allergic response to a defined allergen (122). The authors identified a regulatory immune signature characterized by the *de novo* expression of TNFR2 during early and late stages of AIT whilst all hallmarks of the allergic response were reverted. Particularly, TNFR2 was highly upregulated in GATA3^+^ Tregs as well as in B cells and NK cells. This study showed for the first time that a modified AIT formulation engaging TLR9 activates a TNFR2 program, a critical immune checkpoint for cell homeostasis and cell survival that is known to signal via the non-canonical NF-κB pathway (30, 62).

In an opposite direction, Li and collaborators found that impaired TNF/TNFR2 enhanced Th2 polarization and aggravated allergic airway inflammation in mice (123). Furthermore, the activation of TNFR2 signaling alleviates airway inflammation by decreasing eosinophil and neutrophil recruitment, reduces the expression of pro-allergic cytokines in serum and bronchoalveolar lavage fluid, while at the same time impairs Th2 and Th17 polarization by inhibiting GATA3 and RORγ expression and promoting the expression of FoxP3 and T-bet in CD4^+^ T cells (124). Similarly, agonistic TNFR2 antibodies prevented the loss of FoxP3 expression in Tregs through the consistent hypomethylation of the FoxP3 gene locus. This effect also required the dual use of rapamycin, inferring that FoxP3 expression is dependent both on mTOR and NF-κB (30, 125). In the same line, anti-TNFR2 single-chain variable fragment (scFv) and TNF-α muteins that selectively activate TNFR2 signaling enhanced expansion and function of CD4^+^CD25^+^ Tregs and CD4^+^Foxp3^+^ Tregs (126, 127). Aside from Tregs, the expression of TNFR2 induced by interferon beta has been shown to mark tolerogenic cDC2s with potential to induce Tregs in the lungs and promote mucosal tolerance (128, 129). A patient-based investigation showed differences in the expression of TNFR2 on immune cells between healthy controls and patients with bronchial asthma (130). In summary, emerging evidence indicates that TNFR2 signaling plays a vital role in allergic responses in both, a therapeutic context (122) and in regulating the function of T2 immunity (123, 124).

Venom AIT, the most successful (>90% of clinical efficacy for bee venom; >95% for vespid venom) clinical therapy for allergen tolerance restoration in humans (115, 131), serves as an excellent model to study mechanisms of immune modulation in AIT. An observational clinical study involving patients undergoing ultra-rush venom AIT aimed at characterizing immune responses within the first hours and days after AIT initiation (132). Already 8h after the start of AIT injections, Th2 cells of insect-venom allergic patients showed upregulation of *BCL3* as one of the top 3-regulated transcripts – the gene encoding for BCL3, an atypical IκB protein modulating NF-κB responses (133, 134) mainly by supporting the role of the non-canonical NF-κB signaling pathway – during the induction phase of tolerance-promoting insect-venom AIT. Thus, this study provides evidence that non-canonical NF-κB signaling is one of the first pathways regulated very early on during tolerance-restoring insect venom AIT (132). However, the question remains whether this mechanism is also present in other clinical examples of induced or natural allergen tolerance.

**Figure 4:**
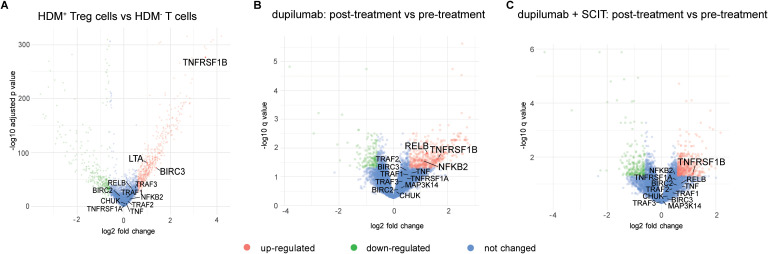
Review of TNFR2 transcriptomic signatures in publicly available allergy treatment datasets. **(A)** Volcano plot showing differentially expressed genes between HDM-reactive Treg (HDM+ Tregs) cells and HDM-non reactive T cells (HDM- T cells) (135). **(B)** Volcano plot showing differentially expressed genes between nasal brushings from patients before and after treatment with dupilumab only (136). **(C)** Volcano plot showing differentially expressed genes between nasal brushings from patients before and after treatment with a combination of dupilumab and SCIT (136).

In this context, we reviewed two recently published data sets for TNFR2 and non-canonical NF-κB signatures, thus uncovering additional information about TNFR2 and its preferred pathway in relevant allergic disease contexts that were not highlighted in the main findings of the original publications. In the first study, Seumois et al. (135) conducted an in-depth analysis of the molecular characteristics of CD4^+^ Th and Treg cells obtained from PBMC of individuals with house dust mite (HDM) allergy and asthma. Transcriptomic analysis (single-cell RNA sequencing) revealed a significant upregulation of the TNFRSF1B (TNFR2), BIRC3 (cIAP2) and LTA (LT-α) genes in HDM^+^ Treg cells (HDM allergen-reactive Treg) compared to T cells non-reactive to HDM (**Figure 4A**), indicating a more pronounced signaling through TNFR2 and corresponding TNFR2 ligands in HDM^+^ Treg cells (135). We reviewed another more recently published dataset of a patient-based study where transcriptomic analysis was applied to investigate the mechanisms of immune tolerance induction in allergic rhinitis (AR) by comparing the effect of various treatments on the local disease signature by sampling nasal brushings from individuals with AR to Timothy grass pollen (136). The treatment arms included the monoclonal antibody dupilumab targeting the common receptor for IL-4 and IL-13 (IL4/IL13Rα), allergen-specific subcutaneous immunotherapy (SCIT), their combination, and placebo. Notably, both dupilumab alone and dupilumab/SCIT treatments resulted in upregulation of TNFRSF1B (TNFR2) gene expression post-treatment compared to pre-treatment nasal allergen challenge (**Figures 4B, C**) whilst all hallmarks of allergic rhinitis were controlled (136).

In the context of therapies for allergic diseases, TNF signaling has been demonstrated to modulate Treg function in allergic asthma (137), positioning the TNF/TNFR2 axis as a possible therapeutic option for atopic diseases (**Figure 3**). Although anti-TNF biologics such as infliximab, adalimumab or etanercept could have an indirect positive effect via TNFR2, these drugs have not been widely adopted as treatments for T2 disease phenotypes due to inconsistent clinical efficacy (138, 139). The TNF-neutralizing monoclonal IgG1 antibodies infliximab and adalimumab could enhance the binding of LT-α to TNFR2 by capturing the majority of available sTNF and tmTNF. On the other hand, etanercept, a fusion protein of the extracellular domain of human TNFR2 and the Fc region of human IgG1, not only neutralizes sTNF and tmTNF, but also LT-α, a TNFR2 ligand with high affinity and activation potential. This interaction may potentially dampen TNFR2 signaling, thereby weakening its immunoregulatory effects and contributing to the lack of sustained therapeutic benefit in allergic patients receiving etanercept (140, 141).

More precise and safer therapeutic approaches with the potential to boost tolerance towards allergens are urgently needed in the allergy field. Undoubtably, novel drugs promoting the expression and activation of TNFR2, such as CpG-ODN and agonistic antibodies, are an avenue to enhance potent tolerogenic Tregs and Bregs with allergen specificity (142). So far, agonistic treatments that target TNFR2 have been exclusively evaluated outside the allergy field (143). In addition, such antibodies have not been pursued in combination with antigen- and allergen-specific tolerance induction. Altogether, the current literature and data on TNFR2 in the context of allergic conditions point to a role of this receptor in keeping allergic inflammation at bay with a potential for therapeutic intervention.

Although less explored than classical AIT, prophylactic AIT treatments that aim to prevent further allergen sensitization by limiting epitope spreading have also been proposed as an effective way to reduce the disease burden caused by allergic diseases in modern societies. Indeed, AIT can also be used at an earlier age to prevent polysensitization, which is a suitable therapeutic option for treating allergic respiratory diseases like allergic rhinitis, asthma, or food allergies at the early stages (144, 145). Gut microbiome-directed interventions provide another important avenue in the development of effector or tolerant immune responses to different antigens, making them a therapeutic and preventive option for the treatment of T2 inflammatory diseases such as asthma or food allergy (146, 147). Within these preventive windows of opportunity for novel treatments of allergic diseases, activating and promoting TNFR2 could be an additional strategy to induce early allergen tolerance by favoring development and expansion of regulatory immune cells.

Interestingly, and to the best of our knowledge, among the other TNF receptor superfamily members that induce predominantly the non-canonical NF-κB pathway, only CD27 (TNFRSF7) might also have a protective role in the context of allergy. It has been shown that pathogenic allergen-specific CD4^+^ T cells are terminally differentiated and lack CD27 expression, whereas AIT induces deletion of these cells (132, 148). On the contrary, some of the other members of the TNF receptor superfamily (CD30, OX40) are involved in the development of allergy and asthma (149–151). Thus, the role of TNFR2 in promoting immune tolerance in the context of allergy is rather unique, probably due to its more prominent expression on Tregs and Bregs.

## 7 Conclusion and perspectives

Allergy and other immune-driven diseases sharing the feature of imbalanced immune responses have become a major health problem worldwide. Therapies that accurately promote immune tolerance in chronic inflammatory diseases are scarce at this moment, thus requiring more research in the coming years. Indeed, this may be partially due to limited knowledge on key cellular and molecular mechanisms that initiate, promote, and maintain immune tolerance towards self- or foreign antigens. A clear example for this notion are allergic diseases, where a disease-modifying and even curative antigen-specific therapy exists in the form of AIT. Despite having gained a deep understanding of the mechanisms of Th2-driven, IgE-mediated allergic diseases over recent decades, we only have an incomplete knowledge on the precise mechanisms driving the early and later switches to peripheral immune tolerance as the basis of clinically successful AIT with sustainable long-term allergen tolerance. For example, multiple AIT studies have demonstrated that grass pollen immunotherapy is successful when administered for three years by either subcutaneous (SCIT) or sublingual (SLIT) route, and all hallmarks of tolerance were observed (152–154). When AIT is performed for only two years, the clinical and immunological outcome was comparable to the three-year outcome, both for SCIT and SLIT (155). However, it was striking that terminating the AIT treatment already after two years and re-evaluating patients without further AIT treatment after three years resulted in loss of tolerance at the three-year-evaluation in both treatment groups (SCIT and SLIT) compared to placebo, as demonstrated by a symptomatic clinical response to allergen challenge at the three-year follow-up (155). This underscores the importance of identifying critical immunological events that link the various levels of allergen tolerance observed during the course of successful long-term AIT. Due to its indisputable anti-inflammatory properties, TNFR2 could fulfil a decisive role in this context as a crucial receptor for restoring allergen tolerance. Thus, further investigating TNFR2 in allergic diseases and AIT might aid to develop safer and enhanced therapies for allergic diseases. Although therapeutic research on targeting TNFR2 in several immune disorders (e.g., in cancer, autoimmunity) has shown considerable advances in recent years, there has been only marginal progress using TNFR2 in allergy-based treatments. As summarized in a position paper by the European Academy of Allergy and Clinical Immunology (EAACI) (156), several candidate biomarkers, including IgE/IgG ratios, basophil activation, and T and B regulatory cell markers, have shown potential for assessing the long-term clinical response at late stage of AIT. However, these markers are still under validation for clinical use. This highlights one of the remaining major challenges in AIT: the lack of validated, time-dependent biomarkers to predict the treatment efficacy. In a venom immunotherapy (VIT) study, researchers have revealed early immune changes, including upregulation of SOCS3, BCL3 (which is downstream in the TNFR2 signaling pathway), and S1PR1 transcripts within 0–8 hours of treatment, alongside the induction of IL-10^+^ Bregs and increased plasma IL-6 in a non-inflammatory context (132). Similarly, AIT selectively depletes pathogenic CD27^-^ CRTH2^+^ Th2 cells while preserving IL-10^+^ CD27^+^ memory cells after 3 years of successful AIT (148). These early and late molecular and cellular changes offer promising novel candidate biomarkers that may enable to monitor and guide AIT better in the future. In addition, new and innovative preclinical and clinical studies should be carried out to assess the impact of targeting TNFR2 to treat allergic diseases while scanning for off-targets and side effects. This approach might have the potential to enhance existing therapies such as anti-cytokine monoclonal antibody treatments or AIT, and to develop novel therapeutic avenues for allergic diseases.
